# Prevalence and the impact of hypogammaglobulinemia in newly diagnosed chronic lymphocytic lymphoma patients

**DOI:** 10.1002/jha2.95

**Published:** 2020-09-01

**Authors:** Namrata Singh, Sarah L. Mott, Grerk Sutamtewagul, Ashley McCarthy, Susan L. Slager, James R. Cerhan, Zuhair Ballas, Brian K. Link

**Affiliations:** ^1^ Division of Rheumatology University of Washington Seattle Washington USA; ^2^ Holden Comprehensive Cancer Center (HCCC) University of Iowa Iowa City Iowa USA; ^3^ Mayo Clinic Division of Biomedical Statistics and Informatics Rochester Minnesota USA; ^4^ Mayo Clinic Division of Epidemiology Rochester Minnesota USA; ^5^ Division of Allergy and Immunology University of Iowa Iowa City Iowa USA; ^6^ Division of Hematology and Oncology University of Iowa Iowa City Iowa USA

**Keywords:** chronic lymphocytic lymphoma, hypogammaglobulinemia, IgA, IgE, survival

## Abstract

**Objective:**

To examine the prevalence of hypogammaglobulinemia in chronic lymphocytic lymphoma (CLL) patients and to test the hypothesis that patients with hypogammaglobulinemia have a distinct clinical profile and outcome.

**Methods:**

Immunoglobulin levels (IgA, IgG, IgM, IgE) were measured in newly diagnosed, treatment naïve banked samples of 150 patients with CLL followed prospectively for outcomes. Cox regression models were used to assess the effects of clinical variables on overall survival (OS).

**Results:**

The median age of the selected CLL cohort was 64 years with a male predominance; 96.2% of the patients were white. Fifty‐nine deaths occurred during a median follow up of 6.8 years. Hypogammaglobulinemia in CLL was common in our cohort with 88 (58.7%, 95% CI: 50.4‐66.6%) patients having a measurable isotype deficiency. The most common Ig deficiency was IgM (44.0%). IgA deficiency or low IgE was associated with higher Rai stages as well as with higher white blood cell counts at presentation. Any immunoglobulin deficiency was not associated with overall survival.

**Conclusion:**

A significant proportion of treatment‐naïve CLL patients had underlying Ig deficiencies – both in isolation and in isotype combinations. Although a deficiency of IgA or IgE was associated with more severe disease at presentation, the impact of this association was mild.

## 1 INTRODUCTION

Chronic lymphocytic leukemia (CLL), the most frequent type of leukemia in adults, is a disease of the immune system characterized by the clonal expansion of lymphocytes in peripheral blood and bone marrow. Patients with CLL are at increased risk of infectious complications, with up to 8‐50% of deaths in CLL being attributable to infections with no evidence of improvement over time in population cohort studies [1, 2, 3, 4]. Hypogammaglobulinemia is among one of various factors accounting for this increased risk of infections in CLL [5]. The prevalence of hypogammaglobulinemia in CLL has been reported in previous studies to range from 20% to 70% [6, 7, 8, 9, 10, 11]. Hypogammaglobulinemia developing after treatment for CLL is well studied but less is known about the frequency and clinical implications of concomitant hypogammaglobulinemia at time of CLL diagnosis.

CLL is a heterogeneous disease with probably several different etiologies all resulting in clonal B cell proliferation.  The two best established CLL prognostic markers are TP53 aberration and the mutational status of the variable region of the immunoglobulin heavy chain (IGHV) gene [12] These markers provide valuable but incomplete information on pace of disease progression, response to certain therapeutic strategies and expected overall survival. Neither marker substantially informs on the relationship of the disease to other underlying immune dysfunction or risk of infectious complications. A further understanding of the heterogeneity of CLL could be of value in providing more personalized therapy with current treatment options, as well as better understanding the biology needed to develop future treatment options.

Hypogammaglobulinemia can be seen in primary immunodeficiencies, for example, in common variable immunodeficiency (CVID), or be secondary to other processes such as malignancy, drug induced, or infectious [13]. A deficiency of an Ig isotype is usually defined by levels below two standard deviations of the mean of a normal cohort. Although routine measurement of IgE is not part of the work up of hypogammaglobulinemia, Lawrence et al [14] have shown that an undetectable IgE (<2 IU/mL) occurs in only 3.3% of the general population in contrast to 75.6% of patients with CVID. Their findings support the routine measurement of serum IgE in the work up of patients with hypogammaglobulinemia.

The University of Iowa/Mayo Clinic Molecular Epidemiology Resource (MER) is an established resource for discovery of biomarkers in lymphoid malignancies and provides a tool to evaluate the significance of hypogammaglobulinemia in newly diagnosed patients with CLL. This prospectively assembled cohort of newly diagnosed patients with lymphoproliferative disease collects baseline biologic specimens and ongoing patient clinical outcomes with sufficient duration of follow‐up to assess relevant associations in a heterogeneous disease with indolent behavior [15].

There is inconsistency regarding the association between hypogammaglobulinemia and survival in CLL with some studies reporting worse survival [6, 9] while other studies finding no evidence of an association [16, 17]. Two contemporary studies evaluated the prevalence of hypogammaglobulinemia in CLL and neither studied the prevalence and impact of low IgE on outcomes in CLL [8, 9]. Therefore, the objective of this study was to examine the prevalence of hypogammaglobulinemia, examining all isotypes, in newly diagnosed CLL patients and to test the hypothesis that patients with hypogammaglobulinemia have a distinct clinical profile and outcome.

**Key Points**

1. Hypogammaglobulinemia in patients with CLL is common but has varying prevalence.
2. There is inconsistency regarding the association between hypogammaglobulinemia and survival in CLL with some studies reporting a worse survival while other studies reported no evidence of an association.
3. We observed a high prevalence of hypogammaglobulinemia in newly diagnosed, treatment naïve CLL patients.
4. Patients with a low IgA or IgE were associated with higher stage CLL and higher WBC count, perhaps hinting at unrecognized biologic variants in this common disease
5. This study further highlights the need to evaluate patients with CLL for hypogammaglobulinemia and manage it when identified. Ig assessment should perhaps be incorporated into all guidelines.
6. When identified, however, this study does NOT support the need for specific counseling or CLL management as a consequence of low Ig levels.

## 2 METHODS

### 2.1 Patient cohort and baseline characteristics

Patients were participants in the ongoing prospective cohort study of patients with lymphoid malignancies from the Molecular Epidemiology Resource (MER) of the University of Iowa/Mayo Clinic Lymphoma Specialized Program of Research. The MER was initiated as an observational cohort study of prospectively enrolled newly diagnosed lymphoma and chronic lymphocytic leukemia patients evaluated at the Mayo Clinic (Rochester, MN) and the University of Iowa (Iowa City, IA) [15]. Lymphoma patients within 9 months of their initial diagnosis, a US resident, and ≥18 years old were eligible to be enrolled into the MER from September 1, 2002 to June 30, 2015. All leukemia cell immunophenotype and pathology analyses were reviewed to confirm each case met criteria for a diagnosis of CLL. All patients fulfilled the 1996 National Cancer Institute working group criteria for CLL [18] in effect at study initiation subsequently updated to fulfill the diagnostic criteria of the international workshop on CLL when published in 2008 [19]. To be eligible for this study, the patients had to be treatment naïve at the time of serum collection. Baseline clinical, laboratory, and treatment data were abstracted from medical records using a standardized protocol. Participants provided peripheral blood serum samples and were systematically followed every 6 months for the first 3 years, and then annually thereafter. Disease progression (ie, requirement for treatment) and deaths were verified through medical record review. Patients’ reports of no disease progression were verified on an annual basis. The cohort protocol was approved by the institutional review boards at the Mayo Clinic and the University of Iowa and written informed consent is obtained from all participants.

IgG/A/M levels were measured using immunoturbidimetric assay whereas the IgE level was determined using electrochemiluminescence immunoassay. The values of normal range used in our laboratory were used to define cut‐offs for low levels – IgG (<700 mg/dL) and IgM (<40 mg/dL). Low IgA was defined as IgA (<70 mg/dL), and based on prior literature [14], low IgE was defined as IgE (<2 IU/mL).

### 2.2 Statistical analysis

Estimates of IgG, IgA, IgM, and IgE deficiency along with 95% exact confidence intervals are reported. The association between Ig deficiencies and clinical factors were evaluated with Wilcoxon rank sum and chi‐squared (Fisher's exact, where appropriate) tests. Survival probabilities were estimated and plotted using the Kaplan‐Meier method. Cox regression models were used to assess the effects of clinical variables on treatment‐free survival (TFS) and overall survival (OS). Time was calculated from biopsy to first active treatment or death due to any cause for TFS and OS, respectively. Patients without the event of interest were censored at last contact. Estimated effects of predictors are reported as hazard ratios (HR) along with 95% confidence intervals. All tests were two‐sided and assessed for significance at the 5% level using SAS version 9.4 (SAS Institute, Cary, NC).

## 3 RESULTS

### 3.1 Patients

From September 2002 through June 2015, 1467 patients with newly diagnosed CLL were enrolled in the MER Cohort. From this cohort, we randomly selected 150 patients enrolled at University of Iowa with pretreatment serum available. Serum samples were collected a median of 1.1 months after diagnosis (range 0‐31.5, with 143 within 12 months of diagnosis). The clinical characteristics of the patients at time of diagnosis are shown in Table 1. The mean age (SD) of the selected CLL cohort was 63.8 (11.0) years with a male predominance (69.3%); 96.2% of the patients were white. Most patients had Rai stage 0 or 1 disease at diagnosis. Twenty‐two percent received active treatment within 6 months of diagnosis. With a median follow‐up of 6.8 years, 48.0% received at least one treatment course and there were 59 deaths. The causes of death were: secondary malignancy unrelated to therapy (n = 10; 16.9%), infection (n = 4, 6.8%), CLL related (n = 15, 25.4%), unable to obtain records (n = 11, 18.6%), and other causes (n = 19, 32.2%).

**TABLE 1:** Baseline characteristics of the cohort with and without any immunoglobulin deficiency

			Any Deficiency		
Covariate	Statistics	Level	No N = 62	Yes N = 88	*P*‐value
Gender	N (Row %)	F	21 (45.7)	25 (54.3)	.47
	N (Row %)	M	41 (39.4)	63 (60.6)	
B Symptoms	N (Row %)	No	55 (42.0)	76 (58.0)	.67
	N (Row %)	Yes	7 (36.8)	12 (63.2)	
Rai Stage	N (Row %)	0	23 (43.4)	30 (56.6)	.11
	N (Row %)	1‐2	35 (45.5)	42 (54.5)	
	N (Row %)	3‐4	4 (20.0)	16 (80.0)	
Initial Treatment	N (Row %)	Active	12 (40.0)	18 (60.0)	.87
	N (Row %)	Observation	50 (41.7)	70 (58.3)	
Age	N		62	88	.73
	Mean		63.1	64.4	
	Median		63.5	64.0	
WBC	N		62	87	.18
	Mean		18.4	27.1	
	Median		14.1	15.1	

### 3.2 Hypogammaglobulinemia at diagnosis in CLL

**FIGURE 1:**
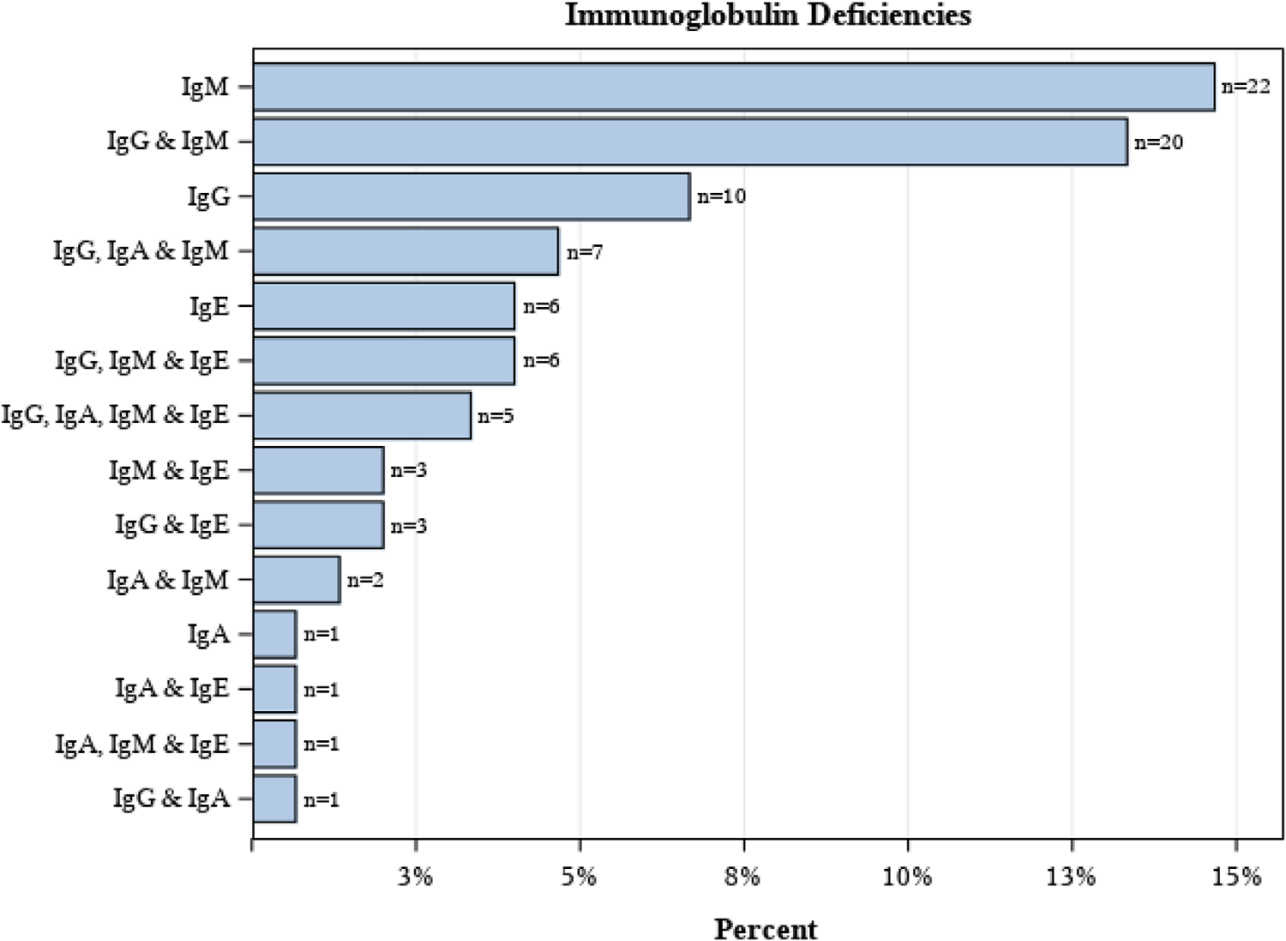
Prevalence of various immunoglobulin deficiencies

Hypogammaglobulinemia in newly diagnosed, treatment‐naïve CLL was common in our cohort with 88 (58.7%, 95% CI: 50.4‐66.6%) patients having a measurable isotype deficiency. No difference in the prevalence of hypogammaglobulinemia was observed based on whether samples were collected ≤12 months versus >12 months from diagnosis (*P* = .24). The most common Ig deficiency was IgM (44.0%) followed by IgG (34.7%), low IgE (16.7%), and IgA (12.0%). Forty‐nine patients had more than one Ig deficiency (Table 2 and Figure 1). The strongest correlation among isotype deficiencies was between IgG and IgM (*ɸ *= 0.43) with the remaining associations ranging from 0.14 to 0.29. The mean and median values for each immunoglobulin subtype is presented in Table 2.

**TABLE 2:** Prevalence of each immunoglobulin subtype in the cohort

Deficiency	Estimate	95% Exact CI
Any	58.7%	50.4‐66.6%
IgG (<700 mg/dL)	34.7%	27.1‐42.9%
IgA (<70 mg/dL)	12.0%	7.3‐18.3%
IgM (<40 mg/dL)	44.0%	35.9‐52.3%
IgE (<2 IU/mL)	16.7%	11.1‐23.6%

### 3.3 Association of hypogammaglobulinemia with clinical presentation and clinical outcomes

Presence of any hypogammaglobulinemia was not associated with gender, stage at presentation, age, or WBC count at presentation, but some differences were seen for specific isotypes (Table 1). IgA (Table 3) deficiency or low IgE (Table 4) were significantly associated with higher Rai stages as well as with higher white blood cell counts at presentation. No association was noted between clinical features at presentation and deficiencies in IgG or IgM (Tables S1 and S2).

**TABLE 3:** Association of IgA deficiency with CLL presentation and treatment

			IgA Deficiency		
Covariate	Statistics	Level	No N = 132	Yes N = 18	*P*‐value
Gender	N (Row %)	F	38 (82.6)	8 (17.4)	.18
	N (Row %)	M	94 (90.4)	10 (9.6)	
B Symptoms	N (Row %)	No	116 (88.5)	15 (11.5)	.70
	N (Row %)	Yes	16 (84.2)	3 (15.8)	
Rai Stage	N (Row %)	0	49 (92.5)	4 (7.5)	**<.01**
	N (Row %)	1‐2	71 (92.2)	6 (7.8)	
	N (Row %)	3‐4	12 (60.0)	8 (40.0)	
Initial Treatment	N (Row %)	Active	28 (93.3)	2 (6.7)	.53
	N (Row %)	Observation	104 (86.7)	16 (13.3)	
Age	N		132	18	.33
	Mean		63.5	66.2	
	Median		64.0	65.5	
WBC	N		132	17	**.02**
	Mean		20.6	45.8	
	Median		14.0	20.7	

**TABLE 4:** Association of IgE deficiency with CLL presentation and treatment

			IgE Deficiency		
Covariate	Statistics	Level	No N = 125	Yes N = 25	*P*‐value
Gender	N (Row %)	F	39 (84.8)	7 (15.2)	.75
	N (Row %)	M	86 (82.7)	18 (17.3)	
B Symptoms	N (Row %)	No	110 (84.0)	21 (16.0)	.53
	N (Row %)	Yes	15 (78.9)	4 (21.1)	
Rai Stage	N (Row %)	0	49 (92.5)	4 (7.5)	**.02**
	N (Row %)	1‐2	63 (81.8)	14 (18.2)	
	N (Row %)	3‐4	13 (65.0)	7 (35.0)	
Initial Treatment	N (Row %)	Active	24 (80.0)	6 (20.0)	.58
	N (Row %)	Observation	101 (84.2)	19 (15.8)	
Age	N		125	25	.74
	Mean		63.9	63.3	
	Median		64.0	63.0	
WBC	N		124	25	**.01**
	Mean		19.1	45.5	
	Median		13.7	20.7	

Median TFS was 6.9 years with 72 (48.0%) patients receiving an initial one course of active treatment. Treatment‐free survival did not significantly differ by hypogammaglobulinemia.

**FIGURE 2:**
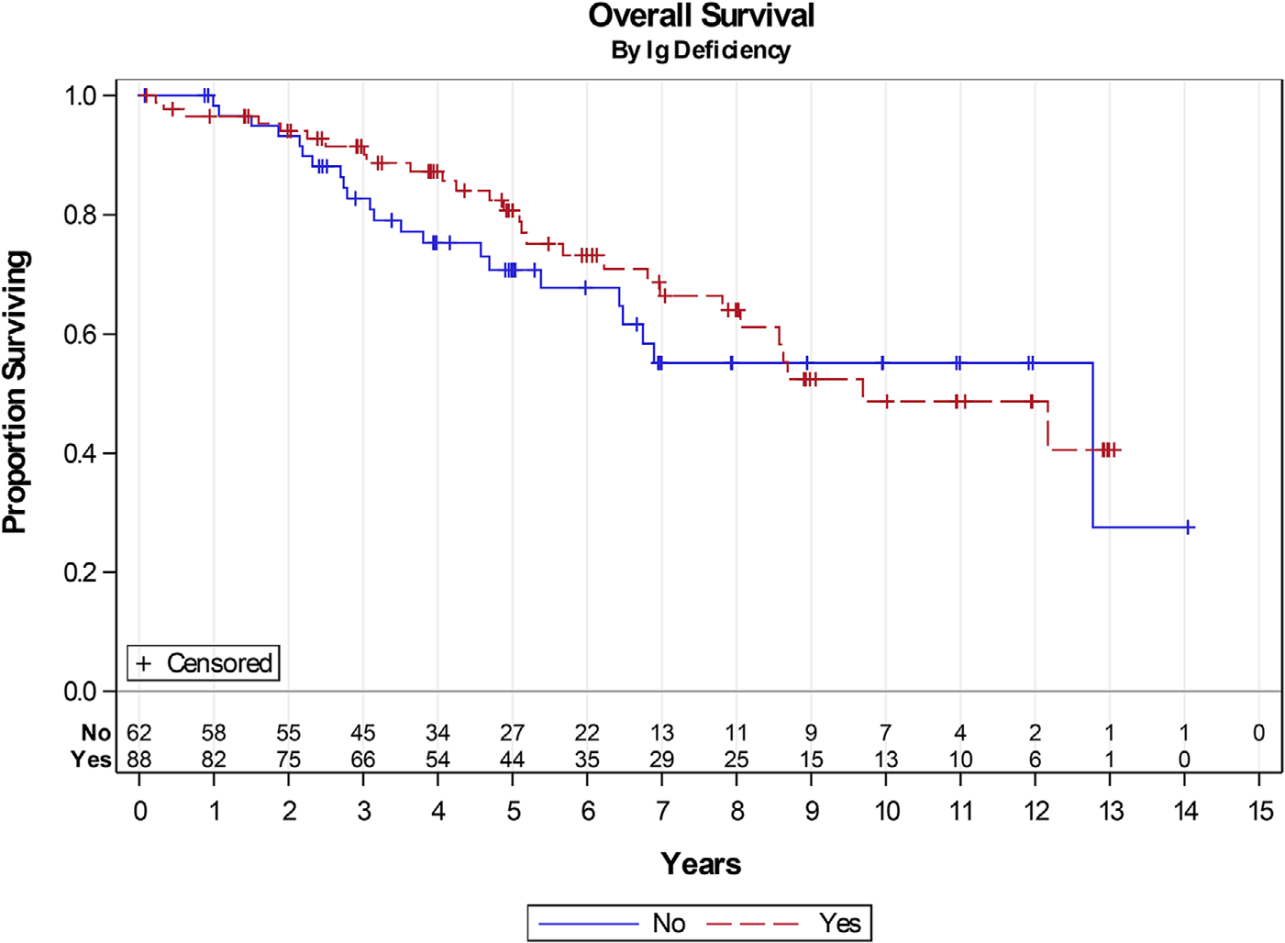
Association of Ig deficiencies with overall survival

Median survival for the entire cohort was 12.7 years with no significant difference between those with any immunoglobulin deficiency (12.2 years) and those with normal immunoglobulin levels (12.8 years; Figure 2). No overall or treatment free survival difference was noted for any specific isotype deficiency (Table S3). Furthermore, multiple deficiencies were not associated with any identifiable difference in clinical outcomes. The specific combination of deficiencies in IgG and IgM – potentially a screening sign for patients with common variable immunodeficiency – was not associated with a difference in overall survival.

## 4 DISCUSSION

We observed a high prevalence of hypogammaglobulinemia in newly diagnosed, treatment naïve CLL patients. Patients with a low IgA or IgE were associated with higher stage CLL and higher WBC count, perhaps hinting at unrecognized biologic variants in this common B‐cell malignancy.

Prior studies, with wide variability in patient populations and few with small sample sizes, have reported prevalence of hypogammaglobulinemia in CLL ranging from 20% to 70% [7, 8, 9, 10]. In another large cohort, Andersen et al found prevalence of hypogammaglobulinemia in at least one Ig isotype (IgG, IgM, or IgA) in 99 of 159 (62%) patients, similar to our study, suggesting this may be close to the true prevalence. A recent study found both reduced and elevated levels of IgG and IgA at diagnosis are important and independent prognostic markers for infection in CLL, with IgA being more relevant as a marker of disease progression and survival [11]. To the best of our knowledge, our study is the first to report on the levels of all Ig isotypes, including low IgE.

There is no consensus regarding the impact of hypogammaglobulinemia on overall survival in CLL (Table 5). This is due to heterogeneous CLL cohorts between studies and the measurement of only single immunoglobulin class in some studies rather than all Ig classes. Andersen et al [9] found any Ig deficiency to be associated with worse overall survival, although Parikh et al [8] with the largest study did not observe any association with OS for any IgG deficiency. In another 1988 study, decreased levels of either IgG or IgA were associated with significantly reduced survival (*P *= .027 and .014, respectively, by Kaplan Meyer curves), but the decreased level of IgM was not (*P *= .46) [6]. Corbingi et al found hypogammaglobulinemia to be the most frequent immunoglobulin aberration present at diagnosis in CLL (13.2%) and IgA deficiency was associated with worse treatment‐free survival compared to patients with normal IgA levels [20]. These contrasting results between different studies highlight that the study of prognostic significance of hypogammaglobulinemia is complex and perhaps modified by several factors. There are also differences in the definitions of hypogammaglobulinemia, the treatment of CLL and management of infection over time and across regions, use of immunoglobulin replacement therapy, and the population and duration of follow‐up in each study. Even our own cohort has now dated patterns of initial therapy as it is unlikely that with the multitude of new therapy options available that any new CLL cohort will have a median TFS of nearly 7 years.

**TABLE 5:** Summary of select few prior studies evaluating association of hypogammaglobulinemia with survival and comparison with current study

Author (year)	Location of study	Hypogammaglobulinemia definition	Prevalence of hypogammaglobulinemia	Median follow‐up	Prognostic impact on OS	Sample size
Ben‐Bassat (1979)	Israel	IgG deficiency (< 620 mg/dl), NORMAL IgM defined as 82±37 mg/dl, IgA 296±129 mg/dl	Low IgG at diagnosis: 18.7%	N/A	No evidence of an association with any Ig deficiency with survival	48
Rozman (1988)	Spain	IgG < 600 mg/dl, IgA < 80 mg/dl, IgM < 50 mg/dl	IgG, 9.9%; IgA, 29.5%; IgM, 30.9%; IgG + IgA, 7.4%; IgG + IgM, 5.1%; IgA + IgM, 13.6%; and IgG + IgA + IgM,4.0%	N/A	Low IgG and low IgA associated with reduced survival (*P *= .027 and .014 respectively)	178
Parikh (2015)	US	IgG < 757 mg/dl	Low IgG 26%	4.2 years (50.4 months)	No evidence of an association of IgG deficiency with survival	1485
Andersen (2016)	Denmark	Deficiency defined as below normal ranges as: IgG 6.1–14.9 g/L, IgA 0.8–4.9 g/L, and IgM 0.41–2.2 g/L	Any Ig 60%; low IgM 50%; IgA 27%; IgG 17%	40 months	Any Ig deficiency associated with shorter OS	159
Ishdorj (2019)	Canada	Normal range: IgG, 6.9 to 16.2 g/L; IgA, 0.7 to 3.8 g/L; and IgM, 0.6 to 2.6 g/L	23% IgG; 16.6% IgA; 58.8% IgM	66 months	Very high IgA (>4 g/L) or low IgA (<1 g/L)associated with Time to first treatment	479 CLL but Ig measured in 364
Current study (2020)	US	IgG (< 700 mg/dL), IgA (< 70 mg/dL), IgM (< 40 mg/dL), and IgE (< 2 IU/mL).	IgM 44.0%; IgG 34.7%; low IgE 16.7% and IgA 12.0%	6.8 years	No evidence of an association of any Ig with survival	150

Abbreviations: Ig, immunoglobulin; N/A, not available; OS, overall survival; US, United States.

We included measurement of IgE levels in our study due to several reasons. Findings by Lawrence et al support the routine measurement of IgE in the work up of patients with hypogammaglobulinemia [14]. They found that an undetectable serum IgE (<2 IU/mL) occurs in only 3.3% (95% CI, 1.9‐5.7%) of the general population. In contrast, an undetectable IgE occurred in 75.6% (95% CI, 65.6‐85.7%) of patients with CVID. Moreover, 91.2% of patients with secondary hypogammaglobulinemia have an IgE >2 IU/mL suggesting that an IgE <2 IU/mL (lower limit of detection) is indicative of a primary humoral immunodeficiency. It has been shown that patients with IgE deficiency have an increased prevalence of multiple immunoglobulin deficits, autoimmune disease, and non‐allergic reactive airway disease compared to patients with normal to elevated IgE levels [21]. There are studies suggesting that very low IgE (≤2 IU/mL; even if found incidentally) should trigger investigation for symptoms of an immunodeficiency and if present, other serum immunoglobulins should be measured [14, 22]. This strategy might help identification of patients with primary immunodeficiency. In our study, we did find that 16.7% of our cohort of CLL patients were low in IgE and it was associated with higher Rai stages as well as with higher white blood cell counts at presentation.

Although the association of pretreatment hypogammaglobulinemia with overall survival is uncertain, most studies including ours show clear association with stage, a marker of disease burden, and possibly duration of having CLL [8, 9, 23] that explained the shorter time to the first treatment in these studies. Hypogammaglobulinemia is likely a consequence of the oncogenic process of CLL since it is rarely found prior to a few years before the diagnosis [24]; however, the exact process is unknown. This seems to be in concordance with the data from multiple myeloma that immunoparesis (deficiency of non‐clonal immunoglobulins) is associated with the stage at the time of diagnosis [25, 26].”

Our study has a few limitations: the study population was predominantly Caucasian, limiting the generalizability of our results to other ethnicities. It is difficult to capture response rates – and consequently progression using rigorous iwCLL response definitions [27] in an observational study, thus we relied on time to treatment as a surrogate measure of short‐term disease behavior and response to therapy. One of the major strengths of this study is the use of the lymphoma molecular epidemiology resource‐ prospective enrollment of consecutive lymphoma patients, classification of pathology diagnoses according to the WHO criteria, availability of banked serum from these patients as well as collection and validation of key outcomes including, retreatment, transformation, new cancers, and cause of death.

Future areas of investigation based upon these findings would evaluate the development of hypogammaglobulinemia over time after diagnosis or treatment as rates of such may vary across use of different treatment modalities. The relationship between hypogammaglobulinemia and infection in CLL patients should be carefully described. If increased rates of infection are found in hypogammaglobulinemic patients, then potential interventions should be evaluated such as Ig replacement.

To conclude, a significant proportion of treatment‐naïve patients with CLL have underlying Ig deficiencies – both in isolation or in combination with different isotypes. Although a deficiency of IgA or IgE was associated with more severe disease at presentation, the impact of the association was mild and we did not overall identify a clinically meaningful use of this finding except to highlight the question on value of early identification with Ig supplementation as a potential strategy to mitigate the common morbidity of infections in these patients. Our findings also highlight the interaction between global immune dysfunction and emergence of a clonal B cell process. The underlying relationship between these two immunologic disorders deserves further study.

## Supporting information

Supporting Information

## Data Availability

De‐identified data are available upon request.
