# Supplementary material for: Prevalence and the impact of hypogammaglobulinemia in newly diagnosed chronic lymphocytic lymphoma patients
Source: EJHaem. 2020 Sep 1;1(2):537–44. doi: 10.1002/jha2.95 (PMC9176078; doi:10.1002/jha2.95)
Supplement: Supplementary file 1 — Supporting Information [file JHA2-1-537-s001.docx]

**Supplemental Table 1**. Association of IgG deficiency with CLL presentation and treatment

|  | | | **IgG Deficiency** | |  |
| --- | --- | --- | --- | --- | --- |
|  | | | **___________________________** | |  |
| **Covariate** | **Statistics** | **Level** | **No**  **N=98** | **Yes**  **N=52** | **P-value** |
| Gender | N (Row %) | F | 28 (60.9) | 18 (39.1) | 0.44 |
|  | N (Row %) | M | 70 (67.3) | 34 (32.7) |  |
| B Symptoms | N (Row %) | No | 86 (65.6) | 45 (34.4) | 0.83 |
|  | N (Row %) | Yes | 12 (63.2) | 7 (36.8) |  |
| Rai Stage | N (Row %) | 0 | 32 (60.4) | 21 (39.6) | 0.45 |
|  | N (Row %) | 1-2 | 54 (70.1) | 23 (29.9) |  |
|  | N (Row %) | 3-4 | 12 (60.0) | 8 (40.0) |  |
| Initial Treatment | N (Row %) | Active | 21 (70.0) | 9 (30.0) | 0.55 |
|  | N (Row %) | Observation | 77 (64.2) | 43 (35.8) |  |
| Age | N |  | 98 | 52 | 0.91 |
|  | Mean |  | 63.6 | 64.4 |  |
|  | Median |  | 64.0 | 64.0 |  |
| WBC | N |  | 97 | 52 | 0.27 |
|  | Mean |  | 19.8 | 30.3 |  |
|  | Median |  | 15.0 | 14.7 |  |

**Supplemental Table 2**. Association of IgM deficiency with CLL presentation and treatment

|  | | | **IgM Deficiency** | |  |
| --- | --- | --- | --- | --- | --- |
|  | | | **___________________________** | |  |
| **Covariate** | **Statistics** | **Level** | **No**  **N=84** | **Yes**  **N=66** | **P-value** |
| Gender | N (Row %) | F | 31 (67.4) | 15 (32.6) | 0.06 |
|  | N (Row %) | M | 53 (51.0) | 51 (49.0) |  |
| B Symptoms | N (Row %) | No | 73 (55.7) | 58 (44.3) | 0.86 |
|  | N (Row %) | Yes | 11 (57.9) | 8 (42.1) |  |
| Rai Stage | N (Row %) | 0 | 31 (58.5) | 22 (41.5) | 0.81 |
|  | N (Row %) | 1-2 | 43 (55.8) | 34 (44.2) |  |
|  | N (Row %) | 3-4 | 10 (50.0) | 10 (50.0) |  |
| Initial Treatment | N (Row %) | Active | 17 (56.7) | 13 (43.3) | 0.93 |
|  | N (Row %) | Observation | 67 (55.8) | 53 (44.2) |  |
| Age | N |  | 84 | 66 | 0.94 |
|  | Mean |  | 63.5 | 64.3 |  |
|  | Median |  | 64.0 | 64.0 |  |
| WBC | N |  | 84 | 65 | 0.22 |
|  | Mean |  | 19.4 | 28.8 |  |
|  | Median |  | 14.6 | 15.7 |  |

Supplementary Table 3.

|  | | | **Treatment-Free Survival** | | | | **Overall Survival** | | | |
| --- | --- | --- | --- | --- | --- | --- | --- | --- | --- | --- |
|  | | | **----------------------------------------------** | | | | **----------------------------------------------** | | | |
| **Covariate** | **Level** | **N** | **Hazard Ratio** | **95% CI** | | **P-value** | **Hazard Ratio** | **95% CI** | | **P-value** |
| Any Deficiency | Yes | 88 | 1.31 | 0.81 | 2.12 | 0.27 | 0.88 | 0.53 | 1.48 | 0.63 |
|  | No | 62 | Ref | - | - |  | Ref | - | - |  |
| IgG Deficiency | Yes | 52 | 0.82 | 0.50 | 1.34 | 0.42 | 0.71 | 0.40 | 1.24 | 0.23 |
|  | No | 98 | Ref | - | - |  | Ref | - | - |  |
| IgA Deficiency | Yes | 18 | 1.52 | 0.82 | 2.83 | 0.19 | 1.36 | 0.67 | 2.78 | 0.40 |
|  | No | 132 | Ref | - | - |  | Ref | - | - |  |
| IgM Deficiency | Yes | 66 | 1.09 | 0.69 | 1.74 | 0.71 | 0.84 | 0.50 | 1.41 | 0.50 |
|  | No | 84 | Ref | - | - |  | Ref | - | - |  |
| IgE Deficiency | Yes | 25 | 1.41 | 0.78 | 2.52 | 0.25 | 0.93 | 0.46 | 1.89 | 0.84 |
|  | No | 125 | Ref | - | - |  | Ref | - | - |  |
| Number of Deficiencies | Units=1 | 150 | 1.06 | 0.87 | 1.29 | 0.58 | 0.93 | 0.73 | 1.17 | 0.52 |
